# Microbial Metabolites: A Sustainable Approach to Combat Plant Pests

**DOI:** 10.3390/metabo15060418

**Published:** 2025-06-19

**Authors:** Somasundaram Prabhu, Rajendran Poorniammal, Laurent Dufossé

**Affiliations:** 1 Department of Plant Protection, Horticultural College and Research Institute, Tamil Nadu Agricultural University, Periyakulam 625604, India; prabhu.s@tnau.ac.in; 2 Department of Agricultural Microbiology, Tamil Nadu Agricultural University, Coimbatore 641003, India; 3 Laboratoire de Chimie et Biotechnologie des Produits Naturels (CHEMBIOPRO), Université de La Réunion, ESIROI Agroalimentaire, 15 Avenue René Cassin, F-97400 Sainte-Clotilde, France; laurent.dufosse@univ-reunion.fr

**Keywords:** microbial metabolites, primary metabolites, secondary metabolites, pest management, biocontrol

## Abstract

With the sustainable increase in agricultural productivity, the need for safer, environmentally friendly pesticide alternatives is also growing. Metabolites of microorganisms (bacteria, fungi, actinomycetes) are emerging as potential bioactive compounds for integrated pest and disease management. These compounds comprise amino acids, carbohydrates, lipids, organic acids, phenolics, peptides, alkaloids, polyketides, and volatile organic compounds. The majority of them have insecticidal, fungicidal, and nematicidal activities. In this review, the classifications, biosynthetic pathways, and ecological functions of primary and secondary metabolites produced by microorganisms are discussed, including their mechanisms of action, ranging from competition to systemic acquired resistance in host plants. The article highlights the importance of microbial genera (viz., *Bacillus* sp., *Pseudomonas* sp., *Trichoderma* sp., *Streptomyces* sp., etc.) in making chemicals and biopesticides for crop defense. We present the possible applications of microbial biosynthesis strategies and synthetic biology tools in bioprocess development, covering recent innovations in formulation, delivery, and pathway engineering to enhance metabolite production. This review emphasizes the significance of microbial metabolites in improving the plant immunity, yield performance, reduction in pesticide application, and the sustainability of an ecological, sustainable, and resilient agricultural system.

## 1. Introduction

Contemporary agriculture has a pressing dual need to increase crop yields and to minimize the environmental impact. Pest and disease pressures rank as one of the leading factors of food loss, causing an annual loss of global food production of between 20 and 40% for major staple crops. First, synthetic chemical pesticides were put up as a defense system against insects, but their prolonged use and overuse are responsible for problems including pesticide resistance, ecosystem pollution, loss of biodiversity, and human and other living system health problems [1]. These developments have opened the door for biologically based strategies—such as biological control, microbial metabolites, pheromones, and juvenile hormone-based methods—to emerge as strong alternatives, combining effectiveness with environmental safety. Among these, microbial metabolites stand out as bioactive compounds produced by microorganisms like bacteria, actinomycetes, and fungi. These compounds are often synthesized as part of the microorganisms’ natural survival strategies, helping them compete for resources, inhibit rival species, or establish symbiotic relationships with hosts. There are many types of plant growth promoters whose function depends on other factors (e.g., genotype, age, development, and stage of growth), creating an impact on yield. The mechanisms of the PGPR can be positive or negative. Some of these compounds, categorized as primary (essential to basic cellular functions) or secondary (produced as a response to a given stimulus), show a wide spectrum of action against a series of agricultural challenges, including pests, plant pathogens, and nematodes [2].

From the functional point of view, microbial metabolites represent the effect of microorganisms within the ecosystem of nature, that is, their important contribution to defense, competition, communication, or symbiosis. They act to control pests through modes of action such as antibiosis, the release of degradative enzymes, the induction of systemic resistance in host plants, disruption of pest development, and direct toxicity [3,4,5]. Several of these compounds have been formulated into biopesticides and are in use as components of more environmentally benign, integrated pest management (IPM) practices [6].

It is anticipated that the microbial biopesticides market will witness substantial growth, primarily due to increasing consumer preference for environmentally sustainable products and stricter regulations on synthetic pesticide use. According to a recent study, the global biopesticides market was valued at USD 6.7 billion in 2023 and is projected to reach USD 13.9 billion by 2028, growing at a compound annual growth rate (CAGR) of 15.9% during the forecast period. Notably, microbial biopesticides constitute over 55% of the global biopesticide market, underscoring their significant role in sustainable agriculture [7]. Modern technological advances in microbial fermentation, metabolomics, genomics, and synthetic biology are also expediting the discovery and utilization of these natural compounds in agriculture [8,9,10].

This review provides a comprehensive understanding of the role and potential of microbial secondary metabolites in plant protection and sustainable agriculture. It discusses their modes of action, ecological functions, and real-world applications, along with recent advances in their production and delivery systems. Additionally, it highlights the potential to enhance their efficacy through cutting-edge biotechnological and synthetic biology approaches.

## 2. Microbial Metabolites

Eukaryotic and prokaryotic microbes inherently produce a diverse array of chemical substances, collectively known as metabolites, which are essential for their survival and role in ecosystems [11]. These compounds are usually small, with molecular weights under 1000 Daltons, and are at the heart of the chemical processes that support microbial metabolism [12]. The full set of these substances produced by a single organism is called its metabolome—a term introduced in 1997 [13]. The metabolome encompasses substances present inside cells, in the surrounding environment (volatile metabolites). Metabolite patterns are intricately linked to an organism’s characteristics; hence their research yields significant insights into microbial behavior and environmental relationships [14,15,16].

The structure and roles of microbial metabolites vary widely, and their presence shifts based on changing conditions. Microorganisms may synthesize, modify, absorb, or release these compounds depending on their environment and physiological state [17]. Generally, metabolites are classified into three types: polar (dissolving in water), nonpolar (not dissolving in water), and volatile. Even with the vast diversity, many structural differences happen at the atomic level rather than creating entirely new forms [18].

Metabolites may be categorized according to their origin and function. They may be generated internally (endogenous) or obtained outside (exogenous) [19]. They are categorized into primary and secondary metabolites based on their function. Primary metabolites are associated with fundamental requirements such as growth, development, and energy metabolism. Examples include amino acids, nucleotides, and organic acids—typically generated during periods of microbial proliferation [20]. A microorganism unable to make a required primary metabolite is called auxotrophic, a condition that can be fatal unless the missing compound is provided externally [21]. Primary metabolites are often consistent across different species [19,22], though they typically appear in low amounts because they are quickly used up. Occasionally, intermediate compounds such as organic acids (e.g., citric acid, malic acid) or amino acid derivatives (e.g., glutamine, ornithine) accumulate and are released into the environment, often as byproducts of microbial metabolism. These metabolites play key roles in the biological control of crop diseases and pests by inhibiting pathogen growth and inducing plant defenses [23,24].

**Figure 1:**
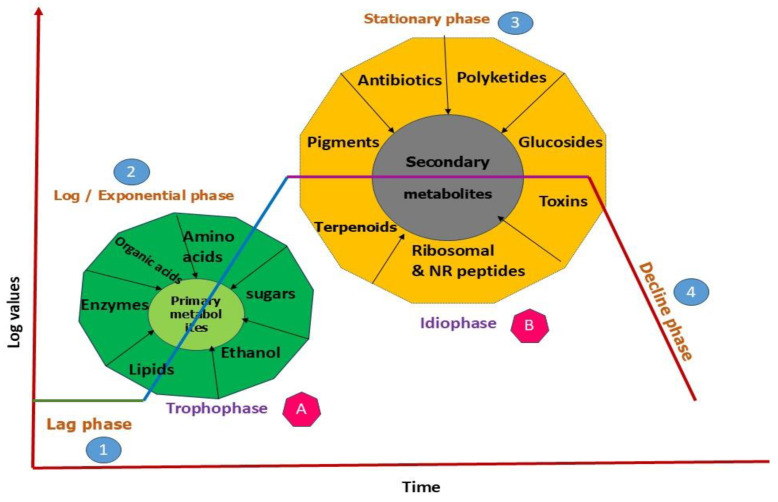
Different phases of microbial metabolites production.

In contrast, secondary metabolites (terpenes, phenolics, peptides, polyketides, etc.) are not essential for immediate survival but provide adaptive advantages for a wide range of microorganisms. They assist in defense, stress reaction, and communication [25]. These chemicals often emerge when cells reach the stationary phase, frequently induced by stress or nutritional deficiencies [26]. Secondary metabolites are often more biologically active in ecological interactions than primary metabolites, as they tend to exhibit targeted effects against pests, pathogens, or competing microbes, making them particularly valuable for biocontrol applications. Although it is helpful to categorize metabolites as primary or secondary, the boundary between them can sometimes shift depending on the situation [19] (Figure 1).

Secondary metabolites have gained significant attention in agriculture due to their potent bioactive properties, many of which have been harnessed as commercial biopesticides. For example, spinosad, a mixture of macrocyclic lactones produced by Saccharopolyspora spinosa, is employed as an effective insecticide in organic farming [27]. Another notable example is abamectin, a secondary metabolite from Streptomyces avermitilis, which acts as a miticide and insecticide by interfering with neurotransmission in target pests [28]. These examples demonstrate the commercial success and ecological potential of microbial and plant-derived secondary metabolites in sustainable pest management.

## 3. Primary Metabolites from Microorganisms

Primary metabolites are small molecules, typically weighing less than 900 Daltons; they are found within cells and play essential roles in fundamental biological processes. They support vital cellular functions such as growth, division, maturation, and reproduction—essentially providing everything a cell needs to function efficiently and maintain its health. Basically, by providing everything a cell needs to be healthy and run effectively, they enable cells to grow, divide, mature, and reproduce. Some well-known examples include amino acids, nucleotides, and fermentation products like ethanol and organic acids. These compounds are absolutely crucial for the growth and survival of microorganisms. Out of all the ways to produce them, microbial production of amino acids stands out because it is highly efficient, cost-effective, and environmentally friendly, offering the added bonus of producing pure enantiomers [29,30] (Table 1).

### 3.1. Amino Acids in Plant Health and Defense

Amino acids are essential for organisms, playing key roles in cellular growth, biofilm formation, and environmental adaptation. They serve not only as building blocks for proteins but also as signaling molecules that support microbial survival and colonization in diverse habitats [31]. These amino acids are grouped by their side chains into three categories: acidic (like aspartic and glutamic acids), basic (such as lysine, arginine, and histidine), and neutral (including serine, threonine, and tyrosine) [32]. Amino acids play a crucial role in the health of both microbes and plants. For plants, they serve as small messengers that direct growth, increase their strength to deal with stress, and help them defend against pests and diseases. A specific amino acid, γ-aminobutyric acid (GABA), allows plants to send signals when they need to activate their defenses. This is especially important during tough conditions or attacks from harmful organisms [33]. Proline, another amino acid, helps maintain cell stability and function in tough conditions [34]. Furthermore, some derivatives of amino acids, like threonine, have natural power to kill harmful microbes. Certain amino acids or their altered forms can also make plants less appealing to herbivores, offering extra protection to the plants [35,36].

**Table 1:** Primary metabolites from microorganisms against plant pests and diseases.

Metabolites	Source Microorganisms	Mode of Action	Target Pests/Pathogens	References
Lipids				
Rhamnolipids (RLs) Fengycins (FGs)	*Bacillus subtilis*	Induce mycelial de-structuring and hyphal fusions	*Botrytis cinerea* and *Sclerotinia sclerotiorum*	[37]
Hormones				
Abscisic acid	*Achromobacter xiloxidans*, *Bacillus pumilus*	Enhances plant resistance	Broad-spectrum fungicide	[38]
Jasmonic acid (JA)	*Pseudomonas*, *Bacillus*, *Azoospirillum*	Induces systemic resistance (ISR)	*Spodoptera exigua*	[39]
Jasmonic acid (JA	*Pochonia chlamydosporia*	PR protein	*Meloidogyne javanica*	[40]
Salicylic acid (SA)	*Pseudomonas*, *Bacillus*, *Azoospirillum*	Induces systemic resistance (ISR)	*Sclerotinia sclerotiorum*	[41]
Ethylene (ET)	*Paenibacillus lentimorbus*	Induces systemic resistance (ISR)	*Sclerotium rolfsii.*	[42]
Indole acetic acid (IAA)	*Dysoxylum gotadhora*	Induces systemic resistance (ISR)	*Verticillium dahliae* and *Fusarium oxysporum*	[43]
Gibberelin	*Rhizobium*, *Bacillus*, and *Penicillium*	Induces systemic resistance (ISR)	All pathogens	[44]
Turanose sugar and hormones IAA, Gibberelic acid (GA) SA	*B. amyloliquefaciens*	Modulation of phytohormone signal	*Rhizoctonia solani*	[45]
Organic acids				
Lactic acid	*Lactobacillus plantarum*	Antibacterial and antifungal	*Pseudomonas campestris*, *Ralstonia solanacearum*, *Xanthomonas campestris* pv. *vesicatoria*, *Pectobacterium carotovorum*	[46]
Lactic acid	*L. paracasei*	Antibacterial and antifungal	*R. solanacearum*	[47]
Organic acid	Most of the microbes	Solubilizes cuticular proteins	Lepidopteran and dipteran pest	[8]
Organic acid	*L. plantarum*	Nematicidal effect	*Meloidogyne incognita*	[48]
Acetic, propionic, formic, benzoic acid	*Lactobacillus* sp.	Interferes with the membrane functions of the pathogen	Broad-spectrum	[49]
Others				
Meso-2,3-Butanediol	*Klebsiella pneumoniae*	Induction of systemic resistance	*R. solanacearum*	[50]
Proteinaceous and non-proteinaceous antifungal compounds	*Lactobacillus plantarum*	Antifungal compounds	*Botrytis cinerea*, *Alternaria solani*, *Phytophthora drechsleri*, *Fusarium* *oxysporum* and *Glomerella cingulate*	[51]

### 3.2. Sugars and Their Role in Pest and Disease Control

Microbes also generate sugar molecules such as oligosaccharides and polysaccharides, which heavily contribute to the enhancement of plant resistance. These sugars act as signaling molecules to induce the plant’s endogenous defense mechanisms against pathogen and insect pests [52]. Some microbial oligosaccharides can increase the synthesis of plant defense enzymes and secondary metabolites, e.g., Šphytoalexins (which retard pathogen growth). Chitosan oligosaccharides [53] and oligogalacturonides [54] obstruct the attachment and invasion by pathogens and deter the feeding of herbivorous insects. In conclusion, microbial sugars are natural agents for eco-friendly pest and disease control.

### 3.3. Organic Acids as Biocontrol Agents

Organic acids, namely those secreted by lactic acid bacteria (LAB), are also fundamental for inhibiting detrimental microorganisms [55]. The most abundant metabolite is lactic acid, although other compounds, including acetic, propionic, formic, benzoic, and polylactic acids, also play a role in the antimicrobial activity of LAB. It has been demonstrated that lactic acid could be used to control the growth of spoilage and pathogenic microorganisms by disorganizing the microorganism’s membrane, reducing intracellular pH levels, and interfering with the metabolic activities, which subsequently leads to cell death [56]. The antimicrobial activity of lactic acid varies depending on LAB species or strain, growth conditions, and microbial consortia [57]. Numerous microorganisms such as bacteria and fungi, are especially sensitive to lactic acid in acidic pH [58].

Some LAB, including *L. sakei*, and *L. curvatus*, synthesize metabolites that act as strong nematicides [59]. Furthermore, compounds produced by *Oenococcus oeni* have demonstrated that they attract spotted wing drosophila and, therefore, could have utility for pest monitoring or behavioral control [60].

### 3.4. Lipids in Microbial Communication and Plant Interaction

A wide range of microbial lipids was produced by an enormous diversity of microorganisms that may be found as intra- or a cell-bound layers, or may be secreted into the surrounding medium [61]. These microbial lipids have important functions in plant–microbe interactions, which include pathogen recognition, signaling, and immunity [62]. Serving as chemical messengers, they control not just interactions between microbes and plants but also those between different groups of microorganisms. During plant defense, microbial lipids can either prime plants for resistance against deleterious invaders or modulate plant response to beneficial microbes [63].

### 3.5. Nucleotides and Their Regulatory Functions

Nucleotides are one of the principal classes of signaling molecules used by microorganisms to signal each other, specifically during quorum signaling, in which the activity group level of a microbial community is regulated. Gene expression is regulated by genes, which are responsible for microbial regulation and secondary metabolite production.

In addition they also contribute to plant defenses. This complex interplay between microbial signaling and plant immune responses highlights the importance of these molecules in maintaining ecosystem health. By facilitating communication within microbial communities and enhancing plant defenses, they play a crucial role in promoting resilience against environmental stressors and pathogens.

## 4. Secondary Metabolites from Microorganisms

Microorganisms are also able to synthesize a wide variety of compounds known as secondary metabolites, generally in the stationary or idiophase stages of growth [26,64]. Primary metabolites are involved in growth and development and are directly associated with essential cellular processes, while secondary metabolites are not critical to the life of the organism. Rather, they contribute to important ecological functions for microbial survival–in competition, defense, signaling, and symbiosis with other organisms [65].

Secondary metabolites, which are often produced during environmental stress or lack of certain nutrients, enable the microbes to cope with the changing environment [66]. Although many of these compounds are well-characterized antimicrobial and bioactive compounds, others remain poorly studied; this is likely due to both the high diversity of microbial species that produce these compounds and the environmental variability experienced [67,68]. They belong to several categories based on their chemical structure, such as antibiotics, toxins, phytohormones, and plant growth regulators [69,70]. Secondary metabolites production is dynamic and dependent upon microbial interactions, environmental signals, and the availability of nutrients [71], and they are commonly grouped into five major structural and functional classes:

- Terpenes (e.g., volatiles, glycosides, sterols);
- Phenolics (e.g., flavonoids, coumarins);
- Nitrogen-containing compounds (e.g., alkaloids);
- Peptides (e.g., surfactin, iturin);
- Polyketides (e.g., 2,4-diacetylphloroglucinol) [72,73,74,75].

Several of these compounds are effective not only as antimicrobials in crops, but also through the induction of host plant disease tolerance, e.g., trichodermin, gliotoxin, and *Bacillus thuringiensis* (Bt) toxin [76,77,78]. These are valuable tools for sustainable agriculture, as shown in Table 2 [79,80].

### 4.1. Terpenoids: Diverse Roles in Plant Protection

Terpenoids, or isoprenoids, constitute the most structurally diverse class of natural products, with over 80,000 compounds identified across all domains of life [99]. These molecules play essential roles in both primary and secondary metabolism and are involved in signaling, defense, and growth regulation. Microbial producers of terpenoids include *Streptomyces* and *Penicillium* sp., which synthesize bioactive metabolites such as erythromycin and antimicrobial agents. A subset of these compounds—microbial volatile organic compounds (MVOCs)—are released into the environment and have been shown to suppress pathogens, promote plant–microbe communication, and stimulate plant defenses [100].

Compounds such as salicylic acid (SA), jasmonic acid (JA), benzothiazole phenol, and pyrazine (as MVOCs) are more powerful in inducing phytoalexin biosynthesis and plant immunity [101]; MVOCs may be a new avenue for inducing plant immunity and an interesting research subject. Such volatiles are greener substitutes for synthetic pesticides and growth promoters [102]. Another group of terpenoids that are essential for fungal growth are the sterols. Although ergosterol is often employed as a fungal biomass marker, it is not present in every fungus [103], thereby highlighting an area that is rarely studied in the breadth of fungal diversity and ecology within the field.

### 4.2. Polyketides

Polyketides are a chemically varied category of secondary metabolites recognized for their potent biological activities, including antibacterial, antifungal, and antiviral properties [104]. Polyketides, synthesized by microorganisms like fungus and actinomycetes, have emerged as crucial agents in agriculture. Examples include avermectins and spinosyns—widely used insecticides that target various insect orders—and the commercial polynactin, effective against spider mites [105,106,107]. Plant-associated Pseudomonas species produce 2,4-Diacetylphloroglucinol (DAPG), another well-studied polyketide, which exhibits a broad spectrum of biocontrol activity [108,109]. This compound not only inhibits the growth of various plant pathogens but also promotes plant health by enhancing root development and nutrient uptake. As research continues, the potential applications of polyketides in sustainable agriculture and pest management are becoming increasingly significant. In addition, the *B. amyloliquefaciens* subsp. plantarum strain produces polyketides like macrolactin, bacillaene, and difficidin, which contribute to its strong antagonistic action against pathogens. These compounds are essential tools for managing plant diseases through natural means.

### 4.3. Phenolic Compounds

Phenolics, particularly flavonoids, are secondary metabolites found in both plants and microbes. These compounds are involved in defense responses, signal transduction, and plant development. Flavonoids are synthesized through the shikimate, phenylpropanoid, and flavonoid-specific pathways [110]. They play key roles in shaping root growth, regulating auxin transport, and ensuring reproductive success in plants like maize and petunia. Their antimicrobial and antioxidant properties make them important contributors to plant resilience under biotic and abiotic stress conditions.

### 4.4. Alkaloids

Alkaloids are nitrogen-containing natural products known for their structural diversity and broad-spectrum bioactivities and are produced by bacteria and fungi. Microbial alkaloids such as indole, isoindole, and ergot derivatives possess antifungal, antibacterial, and nematicidal properties [111]. These properties make microbial alkaloids valuable in agricultural applications, particularly in the development of sustainable pest management strategies. Additionally, their unique structures provide a foundation for the synthesis of novel pharmaceuticals, highlighting the potential of these compounds in both ecological and medicinal contexts. *Epichloë* sp., for instance, produce ergot alkaloids that suppress *Pratylenchus* sp. in the field [112]. *Metarhizium* spp. produce lysergic acid derivatives during insect infection, linking alkaloid production to ecological function rather than plant colonization [113]. Recently discovered alkaloids such as spirobrefeldins from *Penicillium brefeldianum* have demonstrated antimicrobial activity against several pathogens [114].

### 4.5. Peptides

Antimicrobial peptides (AMPs) are small, cationic, and broad-spectrum antimicrobial molecules. These peptides are generally composed of fewer than 50 amino acids and work by disrupting the microbial membranes, and they are effective against many pathogens [115]. AMPs exist in multiple life forms, and serve important functions to combat microbes, defend plants, and regulate immunity [116]. The synergistic action of AMPs, when used in combination, can enhance the plant immune response to a much higher level than that achieved by a single AMP [117].

Peptide antibiotics of a non-ribosomal nature (NRPs), which constitute one subclass of AMPs, are synthesized through large enzyme complexes, termed non-ribosomal peptide synthetases (NRPSs). Others, like *B. subtilis* and *P. fluorescens*, are widely known to produce NRPs, including iturin, surfactin, and fengycin, which are important to plant disease suppression [118] Several NRPs target peptidoglycan synthesis and act as bacteriostatic agents, similar to clinical antibiotics [119,120]. These two peptides as a composite offer a sustainable disease management option in agriculture.

## 5. Mechanisms of Action of Microbial Metabolites Against Insects Pest and Diseases

Microbial metabolites have emerged as potential and eco-friendly alternatives to synthetic pesticides for controlling insect pests and plant pathogens. In contrast to the broad-spectrum chemical insecticides, these bioactive compounds have high specificity in targeting organisms, with limited noxiousness to beneficial species, causing a low ecological risk [121].

Microbial metabolites are particularly useful because they show activity against the host, allowing specific pest control while leaving the related flora and fauna undisturbed. The targeted approach of such a mode of action not only enhances the efficiency of pest management but is also in line with integrated pest management (IPM) and sustainable agriculture.

Although there is a substantial increase in interest in microbial solutions, only a small percentile of microbial diversity has been explored, which is estimated to be just 5% of fungal species and 0.1% of bacterial species globally. This stands to underscore a vast and largely untapped pool of new bioactive agents that could serve as sustainable pesticides and biotechnological and pharmaceutical resources.

Microbial metabolites, which are produced by various microorganisms, including bacteria, fungi, and viruses, are capable of inhibiting or even killing insect pests, as are nematodes that target insects in their natural environment. These metabolites work in different ways, such as by disrupting nerve functions, blocking digestion and reproduction, or boosting the immune responses of the host [25]. In addition, various other microbial strains isolated from marine and terrestrial sediments have been shown to yield secondary metabolites with a great deal of biotechnological significance [122].

Microbial metabolites directly act as pesticides, but they also exert other protective roles such as rhizosphere microbiome modulation, the promotion of systemic resistance in host plants, and exclusion of competing phytopathogens [123]. The broad spectrum of the functions of these roles emphasizes the capability of microbial metabolites as sustainable pest and disease biocontrol agents.

### 5.1. Mechanisms of Action Against Insect Pests

#### 5.1.1. Production of Toxins That Disrupt Insect Physiology

The microbial metabolites are frequently toxins that disrupt normal insect physiology, leading to insect death or developmental dysfunction. Some target the nervous or digestive systems, processes that are essential for maintaining life [124]. One of the common ways of action is through degradation of chitin, which is the main component of the exoskeleton and intestines of insects, leading to impaired structures and gut [125,126].

One of the most characterized microorganisms is *B. thuringiensis* (Bt), which synthesizes Cry and Cyt toxins. These proteins attach to receptors in the gut of the insect, which leads to the lysis and paralysis of the gut, leading to starvation [127]. The specificity of Bt is especially appealing because it reduces collateral damage to less beneficial insect populations and deters environmental risk [128].

#### 5.1.2. Interference with Insect Development and Reproduction

Several microbial metabolites also interfere with hormonal regulation in insects, thereby disrupting development, metamorphosis, or reproduction. For example, decoyinine has been shown to significantly reduce fecundity in small brown planthoppers (SBPH) and to simultaneously alter rice plant physiology, making them less favorable hosts [129,130].

**Figure 2:**
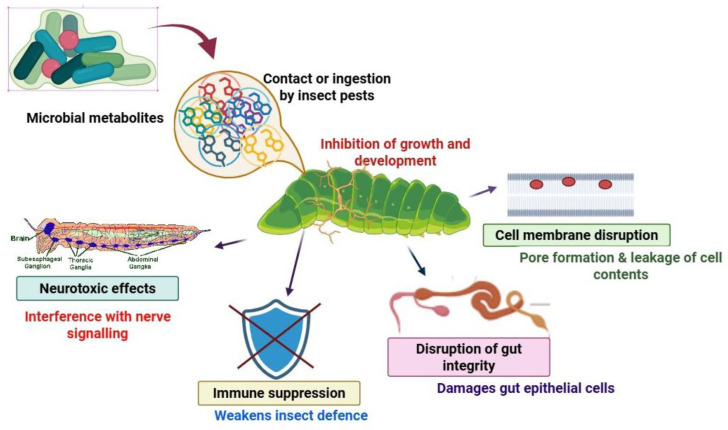
Mechanism of action of microbial metabolites against plant pests (change diagram in Biorender).

Several other metabolites affect the molting process in insects by interfering with the endocrine system, and most notably by mimicking or blocking juvenile hormone (JH) pathways. Nerolidol, as a sesquiterpenoid compound, alters the expression pattern of the JH-degrading enzyme, resulting in modified hormonal levels and disrupted development [131]. Additionally, sakuranetin and other plant-based compounds can diminish populations of yeast-like symbionts that contribute to insect nutrition and stress resistance, thereby compromising pest success (Figure 2) [130].

#### 5.1.3. Repellent and Antifeedant Effects

Microbial VOCs are essential in repelling pests. These compounds can function as repellents or antifeedants, disrupting the perception of insect senses and hindering their ability to detect or exploit host plants [132,133].

Chemosensory and mechanosensory systems are essential for insects to find food and oviposition sites. These ways can be targeted by metabolites that impair pest activity or inhibit feeding [134]. Microbial metabolites provide a sustainable tactic for insect control, either by changing the chemical environment or directly impacting insect receptors.

#### 5.1.4. Disruption of Insect Hormonal Balance

Hormonal disruption is another major part of this mechanism. These microbial metabolites can partially imitate or interfere with insect hormones like the juvenile hormone (JH) and ecdysone, causing developmental stunting, deformities, or even sterility [94,135]. For example, nerolidol simulates JH and alters the expression of JH degradation enzymes, producing hormonal disturbances that break the insect life cycle [131]. This disruption leads to improper molting or metamorphosis in the insect, significantly compromising its reproductive fitness and overall survival.

#### 5.1.5. Induction of Plant Defense Mechanisms Against Diseases

Microbial metabolites also have indirect effects on pest suppression through activation of plant defense systems. Through pattern recognition receptors (PRRs), plants recognize specific pathogen- or microbe-associated molecular patterns (PAMPs/MAMPs), triggering immune responses that lead to systemic acquired resistance (SAR) and/or induced systemic resistance (ISR) [136,137]. Such responses include synthesis of antimicrobial compounds, strengthening of cell walls, and induction of defense genes. Several microbes among the plant’s surrounding rhizosphere also provoke ISR, making the whole plant less susceptible to attacks by insect or microbial problems.

The combined benefits of ISR (defense) and better nutrient absorption suggest that microbes that trigger ISR could be useful in managing pests and nutrients together.

### 5.2. Mechanisms of Action Against Plant Disease

#### 5.2.1. Production of Toxins Against Fungi

Antibiosis is one of the important biological control strategies, through which beneficial microbes secrete antimicrobial compounds to inhibit or damage plant pathogens. These bioactive molecules inhibit crucial metabolic processes, including the integrity of the cell wall, membrane potential, and nucleic acid synthesis within pathogens, ultimately leading to their growth inhibition. For instance, *Trichoderma viride* is known to produce viridiol, an antifungal phytotoxin. Additionally, *Chromobacterium* sp. produces chromobactomycin, a cyclic lipopeptide with antifungal activity [59].

**Figure 3:**
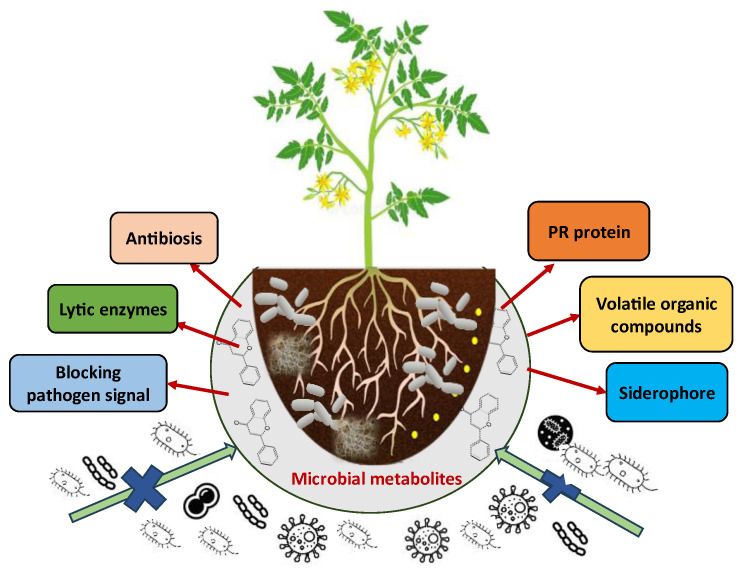
Mechanism of action of microbial metabolites against plant disease.

Many actinomycetes, especially *Streptomyces* species, are known to produce such antimicrobial agents in abundance [138]. In a similar manner, endophytic fungi are among the best sources of antifungal metabolites and have potential against various phytopathogens, such as *Aspergillus niger* and *T. harzianum* isolated from several wild medicinal plants [139]. Such metabolites can be utilized to produce biopesticide product formulations that can control a broad range of plant diseases. Additionally, even some of the antimicrobial metabolites can induce the host resistance responses such as the plant defensive arsenal, from both yield and health perspectives (Figure 3 and Table 3) [140].

#### 5.2.2. Induction of Systemic Resistance in Plants

Microbial metabolites induce plant immune responses by priming through ISR or SAR. These resistances are not mediated by direct contact with pathogens but rather by detecting microbe-associated molecules. After recognition, plants initiate a signaling cascade of events, which involves hormones such as salicylic acid (SA), jasmonic acid (JA), or ethylene (ET), known to trigger the synthesis of defense proteins as pathogenesis-related (PR) proteins [163].

For example, *B. cereus* induces plant resistance pathways via NPR1-dependent signaling, and *P. simplicissimum* and *Paraburkholderia phytofirmans* induce systemic resistance in *Arabidopsis thaliana* against bacterial and fungal pathogens [164].

#### 5.2.3. Enzyme Production to Degrade Pathogen Cell Walls

Lytic enzymes like chitinases, glucanases, proteases, and cellulases break down the structural components of fungal cell walls. These enzymes degrade chitin and β-glucans, major constituents of pathogenic fungal cell walls, thereby weakening and killing the pathogen [165]. Some endophytes from *Fagopyrum esculentum*, such as those in the Bacillus genus, display multiple enzymatic activities and are effective against pathogens like *Fusarium culmorum* and *Rhizoctonia solani* [166]. Their mode of action combines enzymatic degradation with colonization, providing a synergistic approach to disease suppression.

#### 5.2.4. Production of Volatile Organic Compounds (VOCs)

Microbial metabolites are volatile and active metabolites with antimicrobial effects. These alcohols, ketones, terpenes, and sulfur compounds disrupt the pathogen metabolism and membrane structure [167]. In addition, VOCs can induce defense pathways in plants or attract helpful microbes, thus enhancing the aboveground and belowground plant–microbe society network. *Pseudomonas* and *Bacillus* spp. are abundant VOC producers; their emission can be used to suppress soil-borne diseases and promote plant growth [168].

#### 5.2.5. Siderophore Production

Siderophores are iron-chelating molecules that restrict iron availability to pathogens while simultaneously supplying iron to the host plant. This dual function not only suppresses pathogen development but also promotes plant vigor [169,170].

*Trichoderma* and *Pseudomonas* spp. are effective siderophore producers, and their iron-scavenging activity forms a key element of their biocontrol capacity.

#### 5.2.6. Plant Growth Promotion

PGPRs enhance plant growth through a variety of mechanisms: nitrogen fixation, phosphorus solubilization, phytohormone production (e.g., auxins, cytokinins), and indirect pathogen suppression [171]. Species like *Azospirillum* sp. and *Enterobacter* spp. have been shown to increase biomass and yield in rice, sugarcane, and oil palm while also improving plant resistance to diseases such as rice blast [172].

#### 5.2.7. Cell Wall Reinforcement

In response to the microbial elicitors, plants strengthen their cell walls by depositing lignin, callose, and other polysaccharides to prevent pathogen penetration. This signaling network includes the perception of damage-associated molecular patterns (DAMPs) as well as crosstalk between cell wall integrity maintenance and immune signaling [173]. These responses can be intensified, and the structural integrity of host plants can be further strengthened by biocontrol agents such as *Trichoderma atroviride* and beneficial rhizobacteria.

## 6. Mechanisms of Action Against Nematodes

**Figure 4:**
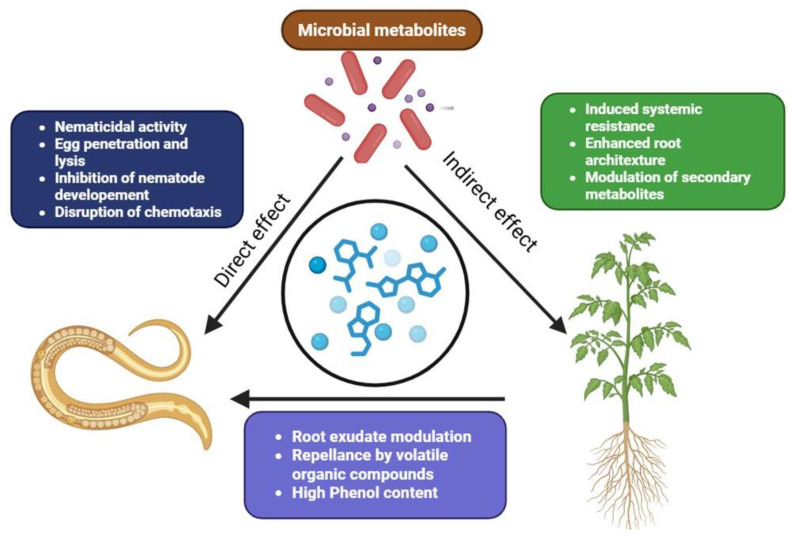
Mechanism of action of microbial metabolites against plant parasitic nematodes.

Plant-parasitic nematodes, particularly root-knot nematodes, threaten global food production by reducing root function and the ability to take up water and nutrients. Microbial metabolites from bacteria, fungi, and other beneficial microorganisms offer an ecologically sound and sustainable alternative to nematicidal chemicals. These organisms reduce nematode populations by different actions, like the production of toxins and enzymes, plant immunity modulation, and alterations in nematode behavior. This section highlights the integrated approaches adopted by microbial metabolites for the effective management of nematode infestations (Figure 4 and Table 4).

### 6.1. Production of Nematicidal Toxins

The primary mechanism by which microbial metabolites control nematodes is through the production of larvicidal or immobilizing toxic compounds. These metabolites, which include both volatile organic compounds (VOCs) and nonvolatile substances, disrupt essential physiological functions within nematodes, affecting their nervous, muscular, and cellular systems. For instance, *B. velezensis* GJ-7 emits volatiles that lead to up to 97% mortality of *M. hapla* juveniles within 48 h [174]. Additionally, *B. firmus* secretes proteolytic enzymes capable of degrading the nematode cuticle, thereby damaging its protective membrane and reducing pathogenicity [175]. These phytotoxins not only directly eliminate nematodes but also hinder their ability to feed, develop, and reproduce, thus offering both immediate and long-term protection to their host plants.

### 6.2. Volatile Organic Compounds (VOCs) as Repellents

VOCs, apart from their toxic potential, can also act as strong repellents which prevent nematodes from coming into the proximity of the plant roots. These compounds disrupt nematode chemosensory activity, leading to an inability to find hosts. For example, *B. amyloliquefaciens* BaNCT02 deterred more than 67% of the juveniles of *M. incognita* from the reproductive structures of the bean roots [173]. VOCs, 2-heptanone and 3-methyl-1-butanol repellents, also inhibit egg hatching, thus providing dual action in the same direction [176]. These repellent effects may contribute to a reduction in nematode colonization and infection.

### 6.3. Induction of Plant Resistance to Nematode Infection

Some microbial metabolites have the capacity to induce the plant’s endogenous defense mechanisms, resulting in systemic resistances. This ISR is mediated by jasmonic acid, salicylic acid, and ethylene signaling pathways. For example, the *B. velezensis* YS-AT-DS1 strain induces JA/SA pathways in tomato plants, thus reinforcing their capability to resist nematodes [177]. Additionally, fungi such as *Trichoderma* spp. can trigger defense reactions, including the production of nematode-repellent compounds and the reinforcement of the cell wall [178,179]. The induced resistance in plants offers long-term protection and decreases reliance on external treatments.

### 6.4. Production of Enzymes That Degrade Nematode Structures

Specifically, enzymes, i.e., proteases and chitinases, are involved in the decomposition of the protective cuticle or eggshell of nematodes. *B. firmus* excrete protease to degrade nematode structural proteins, and *B. wiedmamii* AzBw1 also produce protease and chitinase to attack egg and juveniles, AzBw1 [180].

**Table 4:** Secondary metabolites from microorganisms against plant-parasitic nematodes.

Metabolites	Source Microorganisms	Mode of Action	Target Pathogens	References
Terpenoids				
Abamectin	*S. rochei*	Nematicidal effect	*M. incognita*	[181]
Lactones	*T. harnatum*	Nematicidal effect	*M. incognita*	[182]
Lactones	*Nigrospora* sp. *P. chalmydospora*	Nematicidal effect	*M. incognita*	[183]
Milbemectin	*S.bingchenggensis*	Inhibits the reproduction of nematodes	*M. javanica*	[184]
Dimethyl disulfide (DMDS), methyl isovalerate (MIV)	*Bacillus atrophaeus*	Oxidative stress in nematodes lead to death	*M. incognita*	[185]
Dimethyl disulfide, *S*-methyl ester butanethioic acid	*B. cereus*	Fumigation and repellent activity	*M. incognita*	[186]
Benzeneacetaldehyde, 2-nonanone, decanal, 2-undecanone	*B. megaterium*	Nematicidal effect	*M. incognita*	[187]
3-methoxy-2,5-dimethyl pyrazine,1-undecene, dimethyl disulfide	*P. koreensis*	Nematicidal effect	*M. javanica*	[188]
1-octen-3-ol, 3-octanone	*M. brunneum*	Attraction and kill	*M. hapla*	[189]
1,8-cineole	*Annulohypoxylon* sp.	Nematicidal effect	*Bursaphelenchus xylophilus*	[190]
6-pentyl-2H-pyran-2-one	*Trichoderma* sp.	Nematicidal effect	*B. xylophilus*	[191]
Polyketides				
Bikaverin	*F. oxysporum*	Nematicidal effect	*M. incognita* *Rotylenchulus reiniformis*	[192]
Butyrolactone	*Clonostachys rosea*	Nematicidal effect	*M. incognita*	[193]
Alloaureothin and aureothin	*Streptomyces* sp.	Prevent egg hatching and juvenile mortality	*B. xylophilus*	[194]
2,4-diacetylpholoroglucinol (DAPG)	*P. fluroscens*	Prevent egg hatching and juvenile mortality	*M. incognita*	[28]
2,4-diacetylpholoroglucinol (DAPG)	*P. fluroscens*	Prevent egg hatching and juvenile mortality	*M. javanica*	[195]
Abamectin	*S. avermitilis*	Nematicidal effect	*M. incognita*	[196]
4-heptanone	*Daldinia concentrica*	Nematicidal effect	*M javanica*	[197]
Phenols				
Trichostatin and dehydroxytrichostatin	*S. nigrescens*	Nematicidal effect	*M. incognita*	[198]
Trans cinnamic acid (t-CA) 5-phenylpent-4 enoic acid (PPA) and indole	*Photorhabdus luminescens sonorensis*	Nematicidal effect	*M.incognita* *T. semipenetrans*,	[199]
N-acetyltyramine. benzenepropanoic acid	*Micromonospora* sp.	Prevents egg hatching and juvenile mortality	*M. incognita*	[200]
Napthoquinone Fusarubin	*Fusarium oxysporum*	Affects the nervous system, leading to paralysis	*M. incognita.*	[193]
Nitrogen-containing compounds/Alkaloids				
Alkaloids	*Penicillium bilaiae*	Affects the nervous system, leading to paralysis and death	*P. penetrans*	[201]
Peptides				
Lucinostatin	*Pacilomyces lilacinus*	Prevents egg hatching and juvenile mortality	*M. javanica*	[202]
Rhabdopeptide	*X. budapestensis*	Nematicidal activity	*M. incognita*	[203]
Rhabdopeptides Fabclavines	*Xenorhabdus* sp.	Prevents egg hatching and juvenile mortality	*M. javanica*	[204]
Omphalotin	*Omphalotis olearius*	Disrupts nematode biology	*M. incognita*	[181]

This enzymatic degradation leads to a weakening of the nematodes and increases their susceptibility to the action of environmental stress and other biocontrol agents.

### 6.5. Modulation of Plant Metabolism and Immunity

Microbial metabolites modulate plant hormonal and metabolic pathways to improve robustness. PGPR (plant growth-promoting rhizobacteria), such as *Bacillus* and *Pseudomonas* spp., enhance the production of auxins, gibberellins, and cytokinins, which help in root promotion and nutrient absorption [8]. These hormones, in association with secondary metabolites, such as alkaloids and phenolics, strengthen the plant defense responses against nematode infestation [179]. Moreover, BCAs can also induce the expression of genes that are associated with systemic acquired resistance (ISR) and further enhance the defense capacity of plants at molecular levels [177].

### 6.6. Disruption of Nematode Feeding and Reproduction

Microbial metabolites disrupt the feeding and reproductive behaviors of nematodes and impede nematode development. Enzymes, which are chemicals, can interfere with the nematode’s ability to find the feeding site or mate. For instance, *B. amyloliquefaciens* BaNCT02 and *B. wiedmannii* AzBw1 were effective in decreasing egg hatching and J_2_ emergence through the dual action of VOCs and lytic enzymes [180]. The interruption of these vital life processes reduces the rate and severity of nematode infestation.

## 7. Practical Applications of Secondary Metabolites Against Pests, Diseases, and Nematodes

### 7.1. Microbial Metabolites for Pest Management

Crop protection is essential in modern agriculture to ensure consistent yields and food security. In response, microbial pest and pathogen management has emerged as a promising alternative, offering high ecological safety and strong target specificity. These microbes often exert their pest-control effects through the production of bioactive metabolites that cause pathogenicity or directly kill host pests. Therefore, selecting an appropriate microbial strain for pest management largely depends on the types of metabolites it produces and their specific bioactivity against the target pest [205].

The bacterium *Bacillus thuringiensis* produces a β-exotoxin known as thuringiensin, a thermostable secondary metabolite with insecticidal activity against a wide range of insect orders, including Diptera, Coleoptera, Lepidoptera, Hymenoptera, Orthoptera, and Isoptera, as well as several nematode species [206].

Spinosad, a bacterial metabolite derived from *Saccharopolyspora spinosa*, is another successful example of microbial-based pesticide development for insect control [207]. The insecticidal activity of the metabolites produced by the novel insecticidal bacterium *C.subtsugae* was later confirmed by Martin [208], who discovered the bacterium through his research. Another well-known example is *B.thuringiensis* and its various subspecies, which exhibit insecticidal activity against a broad range of pests, including lepidopterans, coleopterans, and dipterans. These have been widely used in commercial formulations for controlling agricultural pests. Similarly, the toxin complex produced by the enterobacterium *Xenorhabdus nematophilus* has demonstrated insecticidal properties and plays a supportive role in the pathogenicity of its associated entomopathogenic nematodes [209].

Actinomycetes are promising candidates for the development of natural insecticides due to their remarkable ability to synthesize bioactive metabolites. Among these, polyketide compounds represent the primary class of insecticidal agents produced by actinomycetes, including well-known groups such as avermectins, spinosyns, polynactins, tetramycins, and related analogs [107]. Avermectins, derived from the fermentation of *S. avermitilis*, are widely commercialized for crop protection. Their molecular structure enables strong binding to the neuromuscular junctions of certain insects, acting as agonists of γ-aminobutyric acid (GABA)-gated chloride channels, ultimately leading to paralysis and death [210]. These compounds are effective against a wide range of insect orders, including Blattodea, Coleoptera, Diptera, Hymenoptera, Isoptera, and Lepidoptera. Abamectin and emamectin are commercially important insecticidal derivatives of avermectins. Milbemycins, though structurally distinct, share a similar mode of action and are produced by *S. bingchenggensis* [211].

### 7.2. Microbial Metabolites for Disease Management

*Bacillus* spp. possess various antifungal lipopeptides, such as surfactins, iturins, and fengycins, as well as antibiotics, volatile antifungal compounds, and active biomolecules. Such metabolites can enhance their biocontrol potential against plant pathogenic agents [212].

Metabolites and their derivatives isolated from *Streptomyces* spp. have also shown efficacious antifungal activity against different phytopathogenic fungi., e.g., salvianolic acid B can be inhibitory to the mycelia cells and spores of *Alternaria* spp., *Fusarium* spp., *Colletotrichum* spp., *Cladosporium herbarum*, and *Botrytis cinerea*. Another secondary metabolite, 6-amino-5-nitrosopyrimidine-2,4-diol, isolated from crude extract of *Streptomyces amritsarensis* V31 also displayed significant inhibitory potential towards the mycelial growth of various test fungi, like *Rhizoctonia solani* (7.5–65%), *Alternaria alternata* (5.5–52.7%), *Aspergillus flavus* (8–30.7%), *Fusarium oxysporum* f. *lycopersici* (25–44%), *Sarocladium oryzae* (11–55.5%), and *Sclerotinia sclerotiorum* (29.7–40.5%) [213,214].

Bioactive secondary metabolites from actinomycetes were shown to be effective against potato silver scurf disease, induced by the fungus *Helminthosporium solani* [215]. And above all, the efficiencies of producing antifungal metabolites are remarkably high for Streptomyces species. For instance, ansamycin-type antibiotics, including carbamitocins, are also produced by Amycolatopsis CP2808, from which ansamitocin exhibiting marked anticancer activity can be obtained. An endophyte actinomycete *Nocardia* sp. was previously reported as a bioresource for ansamitocin [216].

### 7.3. Microbial Metabolites as Nematode Management

Metabolites produced by actinomycetes—including volatile compounds, fatty acids, hydrogen sulfide, ammonia, alcohols, and phenolic compounds—have been associated with nematicidal activity [217]. Among these, polyketide compounds produced by Streptomyces, such as avermectins and their derivative abamectin, are particularly significant. These metabolites are key components of several widely used commercial biological nematicides. Rashad [218] identified 28 Streptomyces strains exhibiting nematicidal activity against *M. incognita*, potentially linked to their production of enzymes such as proteases, chitinases, and lipases. A nematicidal strain, *Micromonospora* sp. WH06, was isolated and identified. Benzenepropanoic acid (compound 4) exhibited the highest effect, causing 99.02% nematode mortality at 200 μg/mL in 72 h, and it also inhibited egg hatching, highlighting WH06 as a strong biocontrol candidate against *M. incognita* [200].

Volatile organic compounds (VOCs), which are naturally occurring, low-toxicity substances, show promise as biological nematicides. Several fungi are known to produce nematicidal VOCs; for example, *F. oxysporum* emits 2-methylbutyl acetate, 3-methylbutyl acetate, ethyl acetate, and 2-methylpropyl acetate—compounds effective against plant-parasitic nematodes [219]. *Daldinia* cf. *concentrica* produces VOCs that control *M. javanica*, with 4-heptanone identified as the primary active component. Similarly, *Annulohypoxylon* sp. FPYF3050 releases 1,8-cineole, which is highly effective against *Bursaphelenchus xylophilus* [154]. Although many nematicidal metabolites have been reported from nematode-trapping fungi (NTF) VOCs remain less explored. In *Duddingtonia flagrans*, three antimicrobial metabolites—flagranones A, B, and C—have been identified [181].

## 8. Formulation and Delivery Methods for Microbial Metabolites

**Figure 5:**
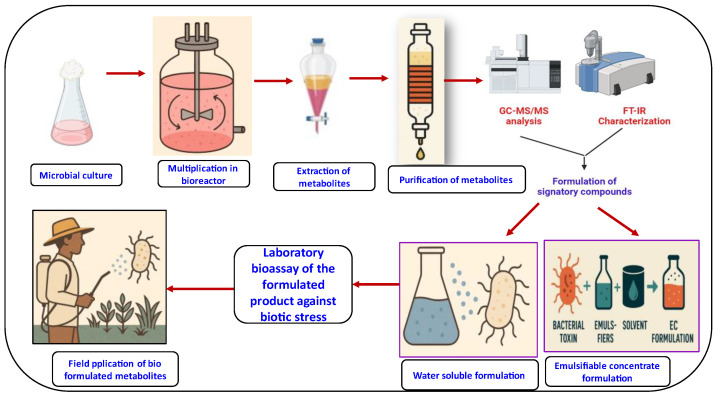
Formulation of microbial metabolites against pests, diseases, and nematodes.

The development of microbial metabolites into biopesticides is an important breakthrough, bringing the use of pesticides in focus for the target-oriented control. A bioformulation is a combination of the biologically active microbial biomass and its metabolites with a carrier. It has eco-friendly uses, including stimulating the growth of plants, improving the uptake of nutrients, and contributing to biocontrol [65]. Bioformulations are of different types, which include solid, liquid, encapsulated, and metabolites and cell-free culture supernatant-based formulations (Figure 5) [220].

Formulations need to be developed in such a way that biocontrol agents are prepared with a sufficient high loading, so that effective and stable control of the target disease is obtained. To do so, the organisms need to be able to survive throughout the different processes of production, transport, storage, and application. Generally, the biocontrol agent is manufactured, formulated, packed, and conserved [221].

Limiting the range of acceptable storage conditions within the distribution channel—such as requiring refrigeration—can restrict market reach. However, improved packaging can help maintain viability by protecting the formulation from fluctuations in moisture and oxygen levels. In many cases, inconsistent conditions can be just as damaging as exposure to extreme temperatures. Repeated freeze–thaw cycles or other significant temperature shifts can harm or kill microbial cells and disrupt cell aggregates [222].

Another critical challenge to preserving high levels of viable spores arises during the application of the biocontrol agent. Formulations must be designed to ensure the organisms remain viable when used with standard farming equipment and stay alive long enough to effectively colonize plant roots or leaves. Some important factors to consider include rain fastness, leaf coverage, and soil penetration. One possible solution is to provide a pilot scale process for a formulation which has been designed to perform well with commonly employed adjuvants and to acquire such information with those adjuvants under different conditions [223].

Stickers may improve the adhesion to seeds and the survival of biocontrol agents and thus can be useful in seed treatments. Knowing the characteristics of the sticker is important; it needs to be effective and easy to apply. For instance, GA is a powerful adhesive, but to canvass it should be heated before use, while some adhesives can be coated at room temperature [224].

Foliar spray formulations should include ingredients that reduce water evaporation and often require adjuvants to protect the active agents from UV light [225]. Low-volume sprays applied at high pressure tend to create fine droplets that dry too quickly, which can prevent the moisture needed for the agent to start infecting the target. Using formulation components with specific properties can help address these issues [226]. Adjuvants can be added to slow down evaporation, improve coverage on leaf surfaces, reduce surface tension from waxes and oils, attract and retain moisture, and form films that help keep the surface moist [227].

## 9. Advances in Biotechnology and Synthetic Biology for Enhanced Metabolite Production

**Figure 6:**
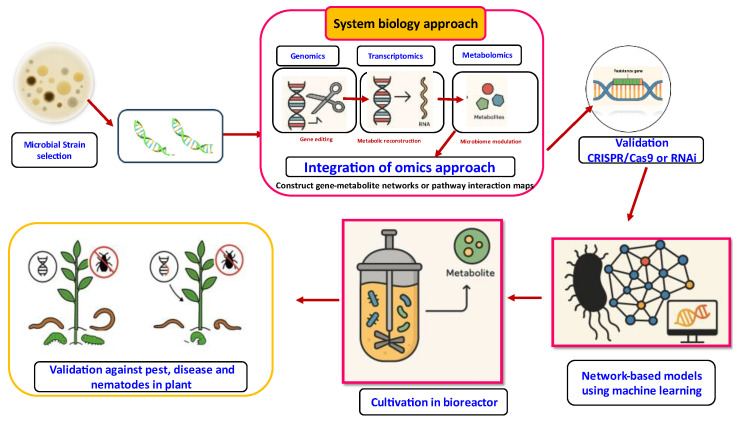
Application of synthetic biology in microbial metabolites for plant health management.

Genetic improvement in microbes is performed by manipulating and improving the strains for enhanced metabolite production and also for improving the efficacy of the metabolites and also for the exclusion of unwanted cometabolites. Microbial strain improvement can be performed by classical genetic methods (including genetic recombination) and by molecular genetic methods (Figure 6) [228]. Classical genetic methods for improvement in microbial metabolites rely mostly on mutation (using both physical and chemical mutagens) followed by rational screening. Rational screening is made for a particular characteristic which is different from that of the final interest but easier to detect. Microorganisms possess regulatory mechanisms that regulate metabolite production and thus prevent overproduction. So the mutants are to be selected for overproduction of desired metabolites. Genetic recombination methods for improvement are by sexual or parasexual cross in fungi and conjugation in actinomycetes and by protoplast fusion in both [229].

There are many methods used for molecular genetic improvement for secondary metabolite production. It is reported that the genes responsible for metabolite biosynthesis are found in clusters in the organisms and which are amplified for higher copies. Molecular improvements can also be made by targeted duplication or amplification of secondary metabolite production genes and amplification of the whole pathway. The negative process of inactivating the competing pathways, silencing the regulatory genes, etc., can also used be in the genetic improvement process [8]. Exogenous manipulation of the microbial genome has increased the production of metabolites and also the effectiveness of biocontrol agents, optimizing their production levels. The expressions of certain genes required for metabolite biosynthesis are enhanced, leading to an augmentation in the formation of compounds of interest. Some of the genes encoding the metabolite degradation are down-regulated, thus preventing the degradation of precious metabolites.

Microbial synthetic biology (SynBio) has been defined as a branch of synthetic biology that uses microorganisms for manufacturing fine chemicals. SynBio in essence integrates and reconstitutes the genetic elements (genes, promoters, transcription factors) from different organisms (source) onto an expression chassis (mostly microorganisms but also advanced organisms have been considered) to produce a certain product of interest [230]. SynBio offers an attractive avenue in this regard, as it allows targeted engineering of prominent microbial strains, thereby unlocking the potential of the microbiome to enhance agricultural sustainability. New, strong strains, such as those found for both of these new species, and root colonizing strains from original communities could be genetically manipulated to increase plant growth without the use of chemical fertilizers and pesticides. This may be a relatively consistent benefit that can be offered by the introduction of plant growth-promoting (PGP) traits to these strains, even as crops change or conditions of culture vary [231].

Microbes engineered to produce antibiotics have also proved their ability to protect plants from biotic stresses [232]. L-threonine transaldolase (LTTA) catalyzed the formation of β-hydroxy-α-amino acids and was essential for the biosynthesis of (2S,3R)-2-amino-3-hydroxy-4-(4-nitrophenyl)butanoate in the plant-associated bacterium *P. fluorescens*. This metabolite has biocontrol properties and is also a precursor for the antibacterial agent obafluorin [232,233].

## 10. Conclusions

The microbial metabolites have potential to be used as environmentally safe pest control agents, providing the multiple effects of protecting the crops and causing negligible environmental harm.

Microbial secondary metabolites can act as an elicitor of plants to enhance the resistance of plants against biotic stress, such as pests, pathogens, and nematodes. They work in two key ways: directly, by generating substances that either damage or kill these threats, and indirectly, by stimulating the plant’s own defense mechanisms. While lab studies with the second greenhouse gas to be recognized show promise, translating those results to growing fields out in the real world can be more challenging. And as environmental conditions—such as temperature and humidity—shift, so too can the ways in which microbes grow and function, resulting in variable outcomes in the field. These are secondary metabolites produced by a variety of microorganisms that have various insecticidal, nematicidal, and antimicrobial activities and thus are excellent candidates for use in integrated pest management (IPM). Compared to conventional pesticides, microbial-based products may currently face higher production costs, scalability issues, and regulatory hurdles, which can impact their commercial viability. For agrochemical companies considering market entry, the cost of production, formulation stability, shelf life, and farmer adoption are critical factors that need to be addressed. Despite these challenges, the growing demand for sustainable and eco-friendly pest control solutions continues to drive research and investment into microbial metabolites, highlighting their transformative potential in IPM strategies.

## Figures and Tables

**Table 2:** Secondary metabolites from microorganisms against plant pests.

Metabolites	Source Microorganisms	Mode of Action	Target Pest	References
Polyketide				
Dihydroxynaphthalene (DHN)-melanins	*Cochliobolus heterostrophus*, *Alternaria*, *Colletotrichum*,	Tolerant to UV-B and reactive oxygen species	Aphid, diamondback moth, beetle, and white fly	[81]
Rugulosin	*Penicillium*, *Phialocephala* *scopiformis*	Induction of programmed cell death	Budworm	[81]
Spinosyn	*Saccharopolyspora spinosa*	Damages nervous system, involuntary muscle contractions, tremors, and paralysis	Lepidoptera, Diptera, Thysanoptera	[82]
Avermectins	*Streptomyces avermitilis*	Disturbances in water balance, molting, metamorphosis, reproductive developments, dysfunction of nervous system	Broad-spectrum insecticide	[51,83]
Stempholone A, Stemphol	*Stemphylium solani*	Insect antifeedant	*Spodoptera littoralis*, *Myzus persicae* and *Rhopalosiphum padi*	[84]
Terpenoids				
2,10-bisaboladien-1-ol	*Alternaria*, *Dydimella*, *Penicillium*, *Fusarium*	Insect antifeedant	*Myzus persicae* and *S. littoralis*.	[85]
Afidopyropen	*Penicillium coprobium*	Weakens the feeding inhibition	Sucking pests	[86]
Strekingmycin, phenalinolactone	*Streptomyces* sp.	Broad-spectrum Insecticide	*Trialeurodes vaporariorum*	[87]
Pyripyropene A	*Aspergillus fumigatus*	Weakens the feeding inhibition	Lepidopteran pests and aphids	[88]
Phenolics				
Stilbenes	*Photorhabdus*	Inhibits the growth of microbes on insect cadavers	Wax moth	[81]
Nucleoside analogs				
Thuringiensin	*Bacillus thuringiensis (Bt)*	Interferes with the RNA polymerase	Diptera, Lepidoptera, Coleoptera, Orthoptera, Hymenoptera, and Isoptera	[89]
Peptide-related compounds				
Fabclavines	*Xenorhabdus* sp.	Anti-symbiotic fungi and bacteria on the insect’s cuticle	Ant	[81]
Destruxins	*Metarhizium anisopliae*	Damages muscular and digestive system	Lepidopteran insects	[90]
Efrapeptins	*Tolypocladium* sp.	Inhibitors of intracellular protein transport	Insecticidal and miticidal effects	[91]
Hirsutellin	*Hirsutella thompsonii*	Inhibits protein synthesis	Aphids, mites, and fruit flies	[92]
Polyoxins and Nikkomycins	*Streptomyces* sp.	Inhibits chitin formation	Broad-spectrum insecticide	[93]
Others				
Crude extract	*Beauveria bassiana*	Insect antifeedant	*S. litura*	[94]
Crude extract	*Xenorhabdus nematophila*	Interferes with host AMPs, insecticidal toxins complex	Lepidoptera, Coleoptera, Diptera	[95,96]
Crude extract	*Serratia entomophila*	Colonization of foregut and cessation of feeding	New Zealand grass grub	[97]
Crude extract	*Chromobacterium subtsugae*	Insect antifeedant	Broad-spectrum insecticide	[98]

**Table 3:** Microbial secondary metabolites for disease management.

Metabolites	Source Microorganism	Target Pathogen	Mode of Action	References
Terpenoids				
Volatile–geosmin	*Streptomyces* spp.	Antibacterial, antifungal	May have allelopathic or inhibitory effects; soil microbes	[141]
Aminoglycosides	*Streptomyces* spp.	Broad spectrum	inhibit protein synthesis by binding to 30S ribosomal subunit	[142]
Viridin	*Gliocladium virens*	Antibacterial, antifungal	Antibacterial, antifungal; inhibits respiration	[143]
Trichodiene	*Trichoderma and Fusarium* spp.	Antibacterial, antifungal	Precursor of toxic trichothecenes; antifungal	[144]
Polyketides				
Koninginin A	*Trichoderma koningii*	Antibacterial, antifungal	Disrupts membrane integrity	[145]
Harzianum A	*T. harzianum*	Antibacterial, antifungal	Induces plant defense response	[8]
Lactone	*T. harzianum*	Antibacterial, antifungal	Disrupts membrane integrity, inhibits conidia germination	[146]
Lactone/butenolides	*T. harzianum*	Antifungal	Inhibits spore germination and hyphal growth	[8]
Lactone/butenolides	*T. harzianum*	Antifungal	Induces systemic resistance	[147]
Lactone/butenolides	*T. harzianum*	Antifungal	Affects cell wall synthesis	[148]
Macrolide	*Streptomyces* spp.	Broad-spectrum bacteria	Inhibits protein synthesis	[142]
Resistomycin	*Streptomyces* spp.	Broad spectrum	Inhibits cell proliferation	[141]
Nitrogen-containing compounds/Alkaloids				
Harzianopyridone	*T.harzianum*	Antifungal	Induces plant resistance	[8]
Gliotoxin	*T. virens*	Antifungal	Immunosuppressive; induces oxidative stress	[147]
Peptides				
Cecropin A	*Hyalophora cecropia*	*F. oxysporum*, *Dickeya dadantii*, *F. verticillioides*	Induces plant resistance	[149]
Iturin	*B. subtilis*	Broad-spectrum inhibitory effect	Interaction with cellular membranes, leading to disruption and subsequent cell death	[150]
Fengycin	*B. subtilis*	*Fusarium*, *Alternaria* and *Botrytis*	Disrupts the integrity of fungal cell membranes, leading to their lysis and subsequent death	[151]
Surfactin	*B. amyloliquefaciens*, *B. subtilis*, and *B. pumilus*	*Fusarium*, *Lasiodiplodia*, *Colletotrichum*, *Botryosphaeria*, *Aspergillus*, and *Penicillium*	Increases the permeability of cell membranes	[152]
Orfamide A	*Pseudomonas* strain	*R. solanacearum*	Direct contact inhibition	[153]
Brevibacillin	*Brevibacillus laterosporus*	*X. campestris* pv. *campestris*	Enhances plant resistance	[154]
Surfactin	*Lysobacter enzymogenes*	*P. syringae* pv. *tabaci*	Enhances plant resistance	[155]
Bacillomycin D (bmyA), fengycin (fenB)	*B. velezensis*	*Ralstonia solanacearum*	Antimicrobial compounds	[156]
Others				
Amphotericin B	*Streptomyces nodosus*	Exhibits antifungal activity	Combats fungal pathogen infections	[157]
Bacillomycin D	*B. amyloliquefaciens*	Antifungal and antibacterial property	Enhances the plant’s defense system	[158]
Cephalosporin	*Acremonium chrysogenum*	Broad spectrum	Inhibits bacterial cell wall synthesis	[159]
Phenazine-1- carboxylic acid (PCA)	*Pseudomonas* spp.	*Phytophthora infestans*	Enhances the plant’s defense system	[160]
Trichokonin VI	*T. psedokoningii*	Broad spectrum	Induces programmed cell death	[161]
Violocein	*C. violaceum*	Exhibits antifungal activity	Enhances the plant’s defense system	[162]
Tyrosol, phenethyl alcohol, 4-hydroxybenzaldehyde	*Curvularia* spp.	*Colletotrichum fragariae*	Enhances the plant’s defense system	[5]

## Data Availability

Not applicable.
